# Two Plastid DNA Lineages—Rapa/Oleracea and Nigra—within the Tribe Brassiceae Can Be Best Explained by Reciprocal Crosses at Hexaploidy: Evidence from Divergence Times of the Plastid Genomes and R-Block Genes of the A and B Genomes of *Brassica juncea*

**DOI:** 10.1371/journal.pone.0093260

**Published:** 2014-04-01

**Authors:** Sarita Sharma, K. Lakshmi Padmaja, Vibha Gupta, Kumar Paritosh, Akshay K. Pradhan, Deepak Pental

**Affiliations:** 1 Centre for Genetic Manipulation of Crop Plants, University of Delhi South Campus, New Delhi, India; 2 Department of Genetics, University of Delhi South Campus, New Delhi, India; New Mexico State University, United States of America

## Abstract

*Brassica* species (tribe Brassiceae) belonging to U's triangle—*B. rapa* (AA), *B. nigra* (BB), *B. oleracea* (CC), *B. juncea* (AABB), *B. napus* (AACC) and *B. carinata* (BBCC)—originated via two polyploidization rounds: a ***U*** event producing the three allopolyploids, and a more ancient ***b*** genome-triplication event giving rise to the A-, B-, and C-genome diploid species. Molecular mapping studies, *in situ* hybridization, and genome sequencing of *B. rapa* support the genome triplication origin of tribe Brassiceae, and suggest that these three diploid species diversified from a common hexaploid ancestor. Analysis of plastid DNA has revealed two distinct lineages—Rapa/Oleracea and Nigra—that conflict with hexaploidization as a single event defining the tribe Brassiceae. We analysed an R-block region of *A. thaliana* present in six copies in *B. juncea* (AABB), three copies each on A- and B-genomes to study gene fractionation pattern and synonymous base substitution rates (Ks values). Divergence time of paralogues within the A and B genomes and homoeologues between the A and B genomes was estimated. Homoeologous R blocks of the A and B genomes exhibited high gene collinearity and a conserved gene fractionation pattern. The three progenitors of diploid *Brassicas* were estimated to have diverged approximately 12 mya. Divergence of *B. rapa* and *B. nigra*, calculated from plastid gene sequences, was estimated to have occurred approximately 12 mya, coinciding with the divergence of the three genomes participating in the ***b*** event. Divergence of *B. juncea* A and B genome homoeologues was estimated to have taken place around 7 mya. Based on divergence time estimates and the presence of distinct plastid lineages in tribe Brassiceae, it is concluded that at least two independent triplication events involving reciprocal crosses at the time of the ***b*** event have given rise to Rapa/Oleracea and Nigra lineages.

## Introduction

The evolution of land plants, particularly angiosperms, has been characterised by repeated rounds of polyploidization [1] and reticulation [2]. Both of these processes appear to have played a major role in the evolution of the Brassicaceae [3], [4]. Taxonomically, Brassicaceae is a well-defined family with a much conserved floral structure. Classification within the family, in contrast, has been contentious and has undergone many revisions [4]–[6]. The most recent taxonomic treatment of the family, which also takes into consideration evidence from molecular systematics, identifies 49 tribes, 321 genera and 3,600 species [6]. Relationship between many taxa, however, remains poorly resolved as a consequence of processes such as convergent evolution and reticulation.

In this study, we focused on evolution within the tribe Brassiceae, which contains some of the world's most extensively grown vegetable and oilseed crops. The most recent polyploidization event in the tribe Brassiceae was described by U [7] based on cytogenetic analysis of three allopolyploid species, *B. juncea* (AABB; 2*n* = 36), *B. napus* (AACC; 2*n* = 38) and *B. carinata* (BBCC; 2*n* = 34), and their three diploid progenitor species *B. rapa* (AA; 2*n* = 20), *B. nigra* (BB; 2*n* = 16) and *B. oleracea* (CC; 2*n* = 18). This round of polyploidy has been termed the ***U*** event [4]. The three allopolyploids behave as strict diploids, in all probability because of a mechanism that suppresses homoeologous chromosome pairing [8], [9].

A more intriguing case of polyploidization is an earlier genome triplication event, referred to as event ***b*** [4], which gave rise to the three diploid *Brassica* species of U's triangle, i.e., *B. rapa, B. nigra* and *B. oleracea*, as well as other species included in tribe Brassiceae. While earlier molecular mapping studies of *B. nigra* suggested the possibility of genome triplication [10], [11], the first clear evidence for this past event came from comparative mapping of the A and C genomes of *B. napus* (AACC) with genic markers from the model crucifer—*Arabidopsis thaliana* [12]. Based on extensive gene collinearity between *A. thaliana* and the A and C genomes of *B. napus* (AACC), the *A. thaliana* genome has been divided into blocks A through X [13]. Each *A. thaliana* gene block is present in at least three copies in each of the two constituent genomes, A and C, of *B. napus* [12]. Similar results have been observed between the A and B genomes of *B. juncea* (AABB) and *A. thaliana* in regard to triplication of each *A. thaliana* gene block [14]. Unequivocal evidence for gene triplication was also uncovered by *in situ* hybridization of *A. thaliana* genomic bacterial artificial chromosomes (BACs) with pachytene-stage chromosome preparations of some key Brassiceae taxa [15].

Sequencing of the *B. rapa* genome has provided strong evidence for genome triplication [16]. The triplication event involving three ancestors (each one with *n* = 7) leading to the formation of a hexaploid (2*n* = 42) was followed by gene fractionation [16], [17], chromosome reshuffling and reduction in chromosome number [18], leading to the evolution of present-day diploid taxa of tribe Brassiceae. Based on gene fractionation patterns, it has been proposed that the hexaploidy was the result of two independent events: two diploid genomes first coming together to produce a tetraploid, followed by crosses with a third diploid species [16], [17]. Early-entry genomes (termed MF1 and MF2) are the most heavily fractionated, with the last genome to enter (designated as LF) experiencing the lowest gene loss [16], [17], [19].

In taxonomic classifications, tribe Brassiceae has generally been considered to be monophyletic based on three conserved morphological traits—conduplicate cotyledons, segmented fruits and simple or no trichomes [5], [6], [20]–[22]. Molecular analysis of the nuclear genome has uncovered evidence of ancient genome triplication across tribal members, supporting a monophyletic origin for Brassiceae [12], [14]–[16]. Analysis of plastid DNA, however, reveals the presence of two distinct lineages—the Nigra (also called Sinapis) lineage and the Rapa/Oleracea lineage [23]–[27]. The presence of these two lineages was first established by RFLP analysis of plastid DNA from various genera and species [23], [24]. These observations have since been confirmed in a number of studies on plastid genes [26] and plastid non-coding sequences [15], [25], [27]. The findings of major studies on plastid DNA divergence confirming the presence of two distinct lineages are summarized in Table S1. The number of distinct lineages has increased to eight through analysis of more number of species [25], [27].

Two competing hypotheses can be put forth to explain the evolution of the *Brassica* A and B genomes: (a) A and B genomes diverged after a single ***b*** event, or (b) A and B genomes arose independently from different reciprocal crosses. Three types of data—gene fractionation patterns, gene block arrangements and synonymous nucleotide substitution rates in homologous genes—are the most pertinent for assessing the validity of these two hypotheses.

In this study, we investigated the genomic structure of an R-block region of *B. juncea* (AABB). The R block is present in triplicate in genomes A (contributed by *B. rapa*) and B (contributed by *B. nigra*). R blocks are gene rich and fairly large [14]. Our initial interest was focused on the R block on linkage group (LG) A10 of *B. juncea*, as it contains some of the most important yield-related quantitative trait loci (QTLs) mapped in *B. juncea* using a doubled haploid (DH) population derived from a cross between the East European gene pool line Heera and the Indian gene pool cultivar Varuna [28]. We aligned genomic BACs in the six R blocks, sequenced the BACs, annotated the sequences and studied gene fractionation pattern in a syntenic region of the R blocks. Pairwise synonymous base substitution rates (Ks values) and divergence times were calculated for paralogous (within A and B genomes) and homoeologous (across A and B genomes) nuclear genes to estimate divergence times of the paralogues and homoeologues. Pairwise synonymous base substitution rates and divergence times were also calculated for the plastid genes *matK* and *ndhF* and compared with the nuclear gene divergence times.

We propose that a hypothesis of reciprocal crosses between progenitor genomes at the time of the entry of the third genome provides the most parsimonious explanation for the presence of two or more plastid lineages in tribe Brassiceae.

## Results

### Identification and sequencing of BACs mapped to the six R blocks of *B. juncea*

In *A. thaliana*, the R block is present as a single block between genes At5g01240 and At5g22030 on chromosome 5 [13]. The six R blocks of *B. juncea* have been previously mapped in DH lines developed from a cross between two lines, Heera and Varuna, from well-defined *B. juncea* East European and Indian gene pools, respectively [14], [29], [30]. The R block of *A. thaliana* is represented in the A genome of *B. juncea* on LGs A2 (∼20 cM), A3 (∼26 cM) and A10 (∼50 cM), and in the B genome on LGs B2 (∼24 cM), B3 (∼29 cM) and B8 (∼47 cM) [14]. Based on examination of gene fractionation patterns, R blocks of A2, A3 and A10 have been respectively identified as most fractionated (MF2), medium fractionated (MF1) and least fractionated (LF) [16]. Previous mapping work using intron-length polymorphism (IP) markers has shown that A2–B2, A3–B3 and A10–B8 R blocks are homoeologous [14].

For analysis of R blocks of the A and B genomes, we focused on a specific region between gene IDs At5g14660 and At5g15840 of the R block of LG A10, which contains major QTLs mapped in a cross between Heera and Varuna [28], [31]. Two BAC libraries developed using *BamH*I- and *Hind*III-digested Heera genomic DNA were used to construct a physical map for all six LGs in the targeted area. This region is syntenous with a 444-kb region of the R block of *A. thaliana*.

BACs corresponding to the above region were identified using region-specific IP markers available on each of the six LGs [14]. A total of 85 BAC clones for the target region in the six R blocks were identified using these markers (Table S2). The gene span of each BAC clone was determined by amplification of genes upstream and downstream of the IP markers used in the initial screening. Once the total gene span of each BAC clone was established, a minimum number of overlapping BAC clones were identified for the targeted region of each of the six R blocks. Contigs on R blocks of A2 and B2 were constructed from four overlapping BAC clones each. Contigs on R blocks of A3 and B3 were created from three overlapping BAC clones each, while contigs on R blocks of A10 and B8 were constructed from five and seven BAC clones, respectively (Figure S1).

A total of 26 BAC clones were sequenced, followed by assembly of BAC-specific contigs. Contigs of the overlapping BACs for each of the six R blocks were assembled into supercontigs (scaffolds) by establishing overlaps. BACs identified for LG A2 and LG B2 were assembled into single scaffolds. BACs of other LGs were assembled into multiple scaffolds. A list of sequenced BAC clones and assembled scaffolds is given in Table S3. A total of 2,470 kb of DNA covering all six R blocks was annotated using the Brassica BAC annotation pipeline (http://brassica.nbi.ac.uk/annotate.html).

### Gene collinearity and fractionation patterns in the sequenced regions of *B. juncea*

Gene contents of the six R block regions of the homoeologous linkage groups A2–B2, A3–B3 and A10–B8 were compared with gene content information available for the R block of *A. thaliana* and syntenous regions of *B. rapa* in the BRAD database (http://brassicadb.org/brad/index.php) (Table S4). The syntenous region of *A. thaliana* on chromosome 5 contains 120 genes. The LF R blocks of *B. juncea* on A10 and B8 have retained about 63% of the genes found in *A. thaliana*, while the MF2 R blocks on A2 and B2 and MF1 R blocks on A3 and B3 have retained about 50% and 43% of these genes, respectively (Table 1). Contrary to the overall trend observed in the genome of *B. rapa* [16], MF2 has more gene retention than MF1 in the region sequenced here.

**Table 1:** Difference in gene content of R blocks in the A and B genomes of *B. juncea*, as compared with the gene content reported in *B. rapa* (A) and *A. thaliana*.

	*A. thaliana*	*B. rapa* MF2-R-A2	*B. juncea* MF2-R-A2	*B. juncea* MF2-R-B2	*B. rapa* MF1-R-A3	*B. juncea* MF1-R-A3	*B. juncea* MF1-R-B3	*B. rapa* LF-R-A10	*B. juncea* LF-R-A10	*B. juncea* LF-R-B8
**Number of ** ***A. thaliana*** ** genes of R block**	**120**	**56** ▴	**61**	**60**	**51** ▴	**53**	**51**	**69** ▴	**75**	**78**
**Number of ** ***Brassica*** ** lineage specific genes of R block**	**-**	**5**	**3**	**2**	**10**	**3**	**-**	**9**	**9**	**6**
* **Number of CDS showing similarity with proteins coded by the ** ***B. rapa*** ** genes from non-collinear blocks**	**-**	**-**	**2**	**13**	**-**	**3**	**2**	**-**	**9**	**14**
* **Number of CDS showing similarity with proteins from genera other than ** ***B. rapa***	**-**	**-**	**3**	**8**	**-**	**1**	**-**	**-**	**-**	**4**
* **Number of CDS showing similarity with transposon related proteins**	**-**	**-**	**-**	**10**	**-**	**1**	**-**	**-**	**1**	**11**

MF2 - Most fractionated subgenome; MF1 - Medium fractionated subgenome; LF - Least fractionated subgenome.

A - A genome; B - B genome; R - R block; LG - linkage group.

CDS - coding sequence.

*- The rows marked with an asterix indicate that the recorded CDS are unique to that LG.

▴ -Difference in gene numbers between *B. rapa* and A genome of *B. juncea* is observed because these genes are present in scaffold sequences of Brassica database (BRAD) but have not been recorded.

**Figure 1:**
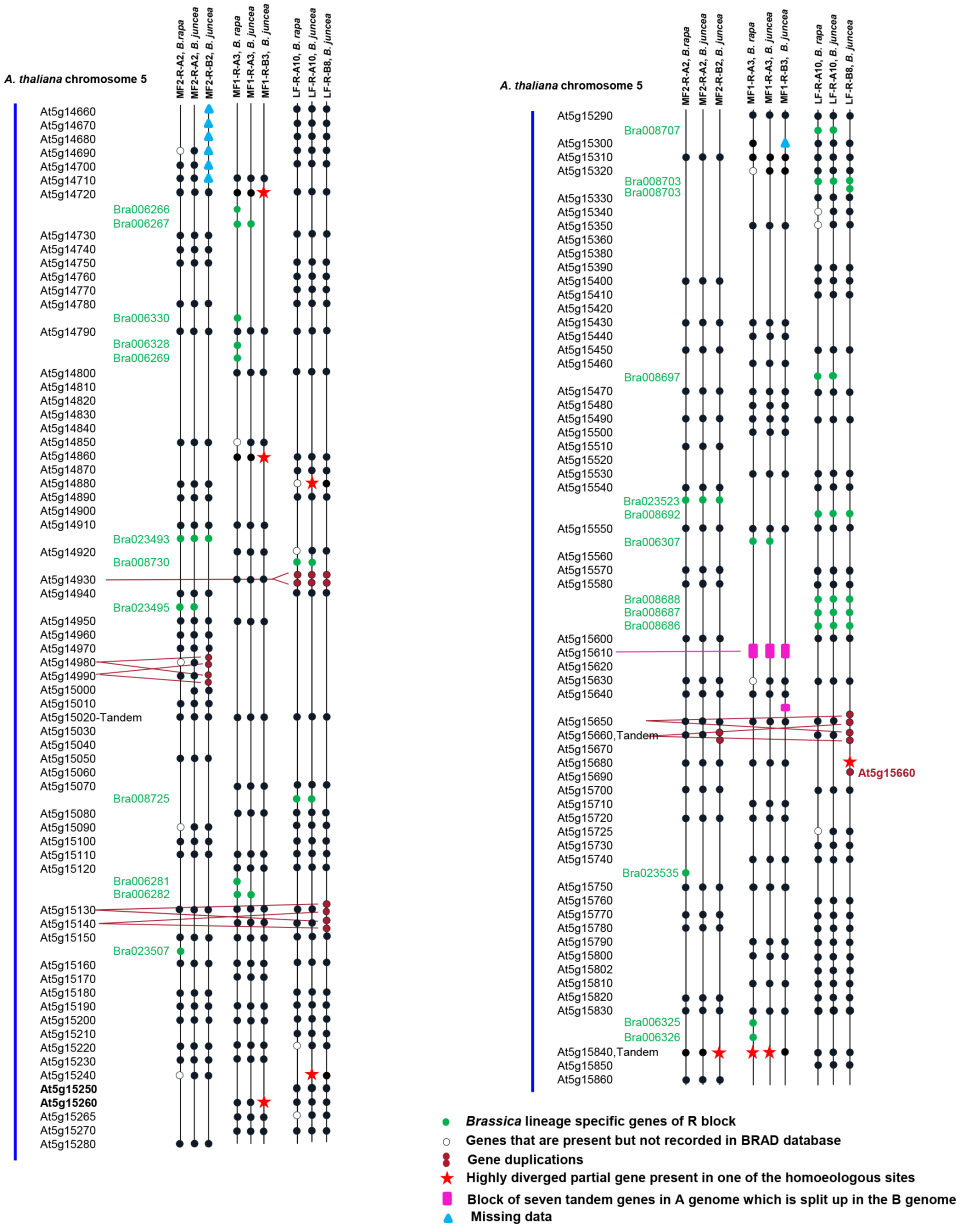
Comparison of the gene organisation in the six R blocks of *B. juncea*. Gene organisation in R blocks of the constituent A and B genomes of *B. juncea* has been compared with the gene organisation in the syntenous region of the R block of *A. thaliana* in chromosome 5 and R blocks of *B. rapa*.

Because intergenic regions are prone to rapid evolutionary changes, we only examined the arrangement and fractionation pattern of coding sequences. Overall, the sequenced region in the A and B genomes of *B. juncea* showed strong collinearity in gene arrangement with that of *A. thaliana* (Figure 1). Sixteen out of 120 genes present in the syntenous region of *A. thaliana* were missing from both A and B genomes of *B. juncea*. The pattern of fractionation between syntenous LGs was identical for 97 of the 104 retained *A. thaliana* orthologues. With respect to the seven other retained *A. thaliana* genes, fractionation patterns differed between A and B genome homoeologues of *B. juncea*: while one of the homoeologues was present as a complete gene, the other was present either as a partial genic fragment or as a sequence showing only low-level of identity. In particular, *A. thaliana* At5g14720, At5g14860 and At5g15260 orthologues were observed in the A genome, while their homoeologues showing low-identity were observed at collinear positions in the B genome. Likewise, At5g14880 and At5g15240 orthologues were observed in the B genome, while their homoeologues in the A genome showed low-identity. At5g15840, a transcription factor-encoding gene, had one orthologue present in MF1 R block of B and another one in MF2 R block of A genome; their respective homoeologues at collinear positions in A and B genomes showed only remnants of identity. The *A. thaliana* gene At5g15680 was absent from the LF block of the A genome, while a partial well conserved sequence of the gene was identified in the LF block of the B genome (Figure 1).

We compared the coding sequence (CDS) content of *B. rapa* available in the BRAD database [32] with predicted CDSs of the *B. juncea* A-genome sequence assembly, and observed a disparity in the number of retained genes (Table 1). However, a BLASTN search for missing genes not recorded in the *B. rapa* BRAD database showed that most were actually present in the *B. rapa* scaffold assembly (Figure 1). High gene collinearity is thus evident between the *B. juncea* A genome and the *B. rapa* genome, in terms of CDS arrangement and the gene content is also similar.

In addition to *A. thaliana* gene orthologues identified in the sequenced region of *B. juncea*, other CDSs were detected by the annotation pipeline. A BLASTN search revealed 23 of these CDSs to be *Brassica* lineage-specific R-block genes. *Brassica* lineage-specific genes from the R block of *B. rapa* described in the BRAD database, i.e., Bra023493, Bra008703, Bra023523, Bra008692, Bra008688, Bra008687 and Bra008686, were observed on homoeologous LGs of both A and B genomes of *B. juncea* (Figure 1). This result is strongly suggestive of A- and B-genome common ancestry. Some *Brassica* lineage-specific genes identified in the A genome of *B. juncea*, i.e., Bra006267, Bra023495, Bra008725, Bra006282, Bra008730, Bra008707, Bra008697 and Bra006307, did not have homoeologues in the B genome (Figure 1). This difference can be attributed to gene losses in the Nigra lineage after the Rapa-Nigra split. A few lineage-specific genes described in the BRAD database, i.e., Bra023507, Bra023535, Bra006266, Bra006330, Bra006328, Bra006269, Bra006281, Bra006325 and Bra006326, were not present in either A or B genomes of *B. juncea*. It is interesting to observe that out of 10 lineage-specific genes on the LG-A3 R block (MF1) of *B. rapa*, only 3 have been retained on LG A3 of *B. juncea*, whereas all 9 lineage-specific genes on the LG-A10 R block (LF) of *B. rapa* are present on LG A10 of *B. juncea*.

A total of 82 CDSs showing no identity with *A. thaliana* orthologues at collinear positions were recognised by the annotation pipeline. These inserted sequences were always specific to a single R block, and appeared as clusters predominantly on LGs B2 and B8. BLASTX comparison of the predicted CDSs with protein databases using a threshold of *E*<e^−5^ revealed significant similarity with protein(s) in the UniProt database. Forty-three CDSs showed similarity with functional proteins encoded by *B. rapa* genes from other blocks, 16 CDSs showed similarity with proteins encoded by genera other than *B. rapa*, and 23 CDSs were associated with transposon-related proteins (Table S5). Transposon-related CDSs were not included in subsequent analyses. A BLASTN search of CDSs against unpublished *B. juncea*, *B. rapa* and *B. nigra* transcriptome databases with an *E*<e^−10^ threshold revealed that 62% were represented in the *B. juncea* transcriptome database. More than 80% of the CDSs expressed in the transcriptome were highly divergent from *B. rapa* gene transcripts and appeared to be ancient insertions. Most of these insertions were observed in the B genome, with a few specific to the A genome. The remaining 20% of CDSs were mostly *Brassica* lineage-specific genes or gene fragments that appear to have recently transposed from noncollinear regions. Most of these transpositions were specific to the A genome. It can be concluded that the A and B genomes of *B. juncea* contain many unique CDSs. This situation may be due to either loss of these CDSs from homoeologous sites after genome triplication or to divergence in the progenitor species contributing to evolution of the A and B genomes.

An interesting feature is the tandemly duplicated nature of several genes present in single copies in *A. thaliana* (Figure 1). Single-copy genes At5g14930 and At5g15610 were found to be present as tandem repeats in both the A and B genomes of *B. juncea* and the *B. rapa* genome. *Arabidopsis thaliana* gene At5g14930 showed tandem duplication on the LF blocks of LGs A10 and B8, while At5g15610, a proteasome component domain protein, was present in seven tandem copies on the MF1 block of LG A3; this latter gene also occurred in four copies on LG B3, although the fourth copy was found to have separated from the other three (Figure 1). Gene-pair duplications specific to the B genome of *B. juncea* were also observed: At5g14980-At5g14990, At5g15130-At5g15140 and At5g15650-At5g15660. In addition to the At5g15650-At5g15660 duplicated gene pair, a third copy of At5g15660 was observed on the LF R block of LG B8 (Figure 1).

### Nuclear genome divergence

Nuclear genome divergence was studied by estimating the synonymous base substitution rates (Ks) for 11 genes (Table S6) retained in all six R blocks of *B*. *juncea* and their respective orthologues from *A. thaliana*. For each of the 11 genes, six CDSs of *B. juncea* annotated by the Brassica BAC annotation pipeline were aligned with *A. thaliana* sequences using the CLUSTAL W codons option of the Alignment explorer in MEGA5 [33]. After removing gaps and codons with ambiguous bases, only common aligned regions from all the sequences were considered for further analysis. Pairwise comparisons were used to estimate Ks values between *B. juncea* and *A. thaliana* genomes (orthologue divergence), and within A and B genomes (paralogue divergence) and between A and B genomes (homoeologue divergence) of *B. juncea*. Divergence times were calculated assuming a mutation rate of 1.5×10^−8^ substitutions per synonymous site per year [21].

**Figure 2:**
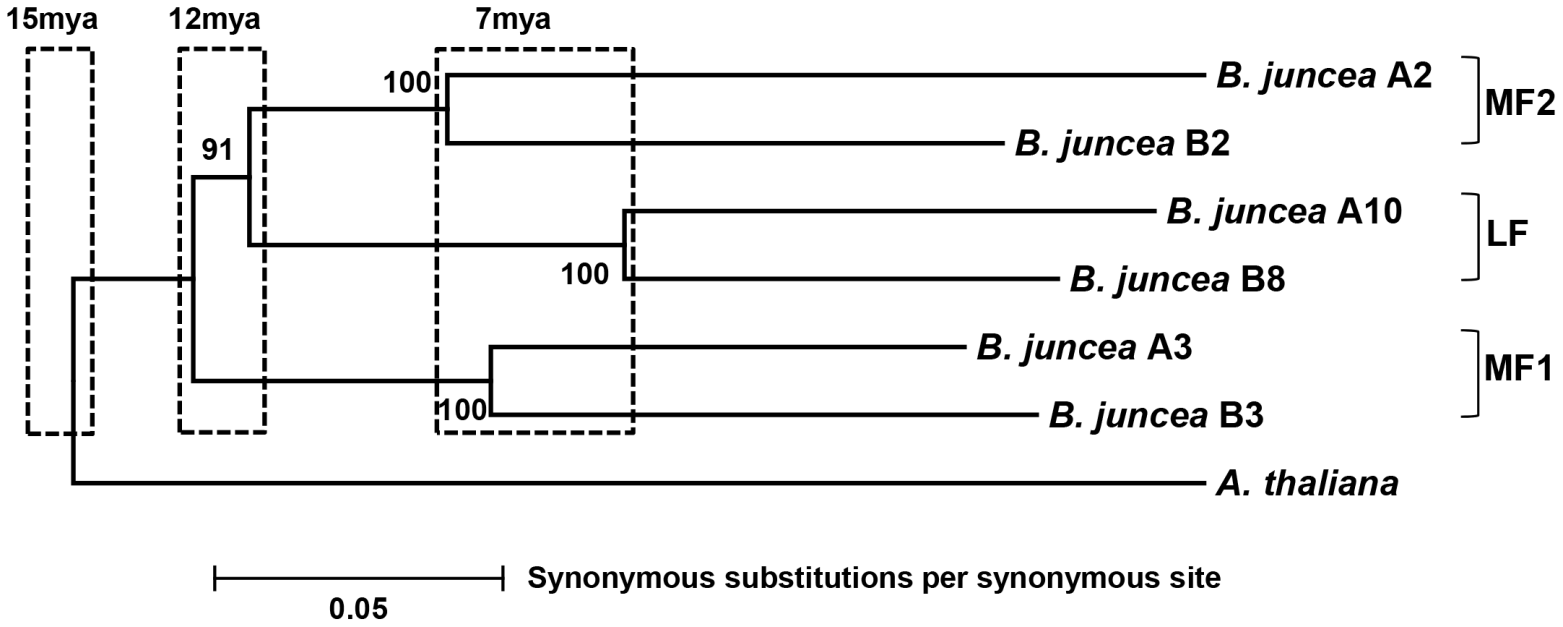
Neighbour-joining tree constructed on the basis of distances estimated from synonymous base substitutions of the concatenated nucleotide sequences of 11 genes retained in all the six R blocks of *B. juncea*. The numbers on the nodes represent percentage from bootstrap analysis of 1000 replicates. *A. thaliana-Brassica* divergence time ∼15 mya; *Brassica* A genome paralogue and B genome paralogue divergence time ∼12 mya; A and B genome homoeologue divergence time ∼7 mya.

Average Ks values for *A. thaliana* (AT)-*B. juncea* (BJ) ranged from 0.43±0.1 (for AT-BJ A3) to 0.49±0.25 (for AT-BJ A2) on the A genome, and from 0.45±0.15 (for AT-BJ B3) to 0.46±0.14 (for AT-BJ B8) on the B genome (Table 2). The mean synonymous base substitution rate, 0.46±0.15, calculated for *Arabidopsis*-*Brassica* indicates that the two genomes diverged about 15 mya (Figure 2). Analysis of variance showed no significant difference (*F*[5,60]  = 0.17; *p* = 0.97) among Ks values derived from the different *Arabidopsis*-*Brassica* comparisons, indicating that all six subgenomes are almost equally diverged from *A. thaliana*.

**Table 2:** Nuclear genome divergence analysis based on synonymous nucleotide substitutions in the genes retained across all the six R blocks of *B. juncea* and their orthologues from *A. thaliana*.

Orthologue divergence			Paralogue divergence			Homoeologue divergence		
Between *A. thaliana* and *B. juncea*			Within the A and B genomes of *B. juncea*			Between the A and B genomes of *B. juncea*		
	Mean Ks value	Divergence time (mya)		Mean Ks value	Divergence time (mya)		Mean Ks value	Divergence time (mya)
A genome			A genome			11 homoeologues		
AT-BJ A2	0.49±0.25	16.3±8	BJ A2-BJ A3	0.36±0.14	11.9±5	BJ A2-BJ B2	0.27±0.14	8.9±5
AT-BJ A3	0.43±0.10	14.5±3	BJ A2-BJ A10	0.39±0.12	13.1±4	BJ A3-BJ B3	0.18±0.07	6.1±2
AT-BJ A10	0.48±0.12	15.9±4	BJ A3-BJ A10	0.36±0.15	12.0±5	BJ A10-BJ B8	0.19±0.08	6.5±3
B genome			B genome			All homoeologues		
AT-BJ B2	0.45±0.12	15.2±4	BJ B2-BJ B3	0.33±0.08	11.0±3	BJ A2-BJ B2	0.23±0.09	7.6±3
AT-BJ B3	0.45±0.15	15.4±5	BJ B2-BJ B8	0.34±0.13	11.2±4	BJ A3-BJ B3	0.21±0.07	6.8±2
AT-BJ B8	0.46±0.14	15.9±4	BJ B3-BJ B8	0.38±0.13	12.6±4	BJ A10-BJ B8	0.21±0.06	6.9±2
**Overall Mean**	**0.46±0.15**	**15.3±4**		**0.36±0.12**	**12.0±4**		**0.21±0.10**	**7.1±3**

Divergence time was calculated by assuming a mutation rate of 1.5×10^−8^ synonymous substitutions per site per year [21].

Overall mean values were calculated from the comparisons made for the 11 genes retained in all the six R blocks.

To decipher the divergence order of the three paralogues within the A and B genomes of *B. juncea*, mean Ks values were estimated by pairwise comparison of genes from these regions. Average Ks values ranged from 0.36±0.14 (for BJ A2-BJ A3) to 0.39±0.12 (for BJ A2-BJ A10) within the A genome, and from 0.33±0.08 (for BJ B2-BJ B3) to 0.38±0.13 (for BJ B3-BJ B8) within the B genome (Table 2). No significant variation was found among Ks values calculated from pairwise comparisons of paralogues on the A genome (*F* [2], [30]  = 0.27; *p* = 0.77) and B genome (*F* [2], [30]  = 0.54; *p* = 0.58), indicating that the three progenitors contributing to the A and B genomes diverged at the same time, about 12 mya (Figure 2). The mean Ks value of 0.36±0.12 (*n* = 66) obtained from paralogue comparison within A and B genomes is significantly different (*p*<0.0001, unpaired Student's *t*-test) from the mean of 0.46±0.15 (*n* = 66) calculated from *Arabidopsis*-*Brassica* comparisons. These results indicate that the three progenitors of *Brassica* A and B genomes diverged after the *Arabidopsis*-*Brassica* split.

To examine divergence of the A and B genomes of *B. juncea*, the rate of synonymous substitutions was calculated for homoeologous gene pairs. Average Ks values ranged from 0.18±0.07 (for BJ A3-BJ B3) to 0.27±0.14 (for BJ A2-BJ B2) (Table 2) with no significant variation (*F* [2], [30]  = 2.2; *p* = 0.12). The mean Ks value of 0.21±0.1 (*n* = 33) calculated from the homoeologue comparisons implies a divergence time of approximately 7 mya for the A–B genome split (Figure 2). This calculated Ks value is significantly different (*p*<0.0001, unpaired Student's *t*-test) from the mean of 0.36±0.12 (*n* = 66) derived from comparisons of paralogues within the A and B genomes. This difference suggests that the divergence of the three paralogues of A and B genomes has more antiquity than the divergence between homoeologues of A and B genomes i.e the divergence of *B. rapa* and *B*. *nigra*.

We constructed a neighbour-joining tree taking into consideration the evolutionary distances estimated from synonymous base substitution rates, using a 9,399-bp long data set comprising concatenated sequences of the 11 genes (Figure 2).

The above-described homoeologue divergence analysis between A and B genomes considered only genes retained across all six R blocks. We carried out an additional analysis that included every possible homoeologous gene pair i.e. 44 gene pairs for BJ A2-BJ B2, 45 for BJ A3-BJ B3 and 54 for BJ A10-BJ B8. Average Ks values ranged from 0.21±0.1 for BJ A3-BJ B3, BJ A10-BJ B8 and 0.23±0.1 for BJ A2-BJ B2 (Table 2). Even with the larger number of genes included in this analysis, the results were similar to the previous analysis, with no significant variation observed in Ks values (*F*[2,140]  = 1.2; *p* = 0.30) between the three homoeologous LGs.

Pairwise divergence was estimated for seven *Brassica* lineage-specific genes present in the homoeologous LGs—two from MF2 (BJ A2-BJ B2) and five from LF (BJ A10-BJ B8) (Figure 1). Calculated Ks values ranged from 0.12 for Bra008703 to 0.23 for Bra023493, with a mean of 0.19±0.04, almost identical to estimated levels of divergence between homoeologous gene pairs of A and B genomes. This result indicates that these genes were present in the progenitors before the A-B genome split.

The antiquity of *Brassica* tandem duplications was studied by calculating pairwise divergence levels between the duplicated gene copies. A Ks value of 0.6 was estimated between copies of At5g14930, a tandemly duplicated gene in both BJ A10 and BJ B8; this value was much higher than the Ks value of 0.2 calculated for the corresponding homoeologues (i.e., Ks between A genome and B genome copies). This result suggests that the gene duplication predated the divergence of A and B genomes and occurred in the progenitor(s) contributing to the ***b*** event. Orthologues of *A. thaliana* genes At5g14980 and At5g14990 are present as tandem duplicates on LG B2, and At5g15130, At5g15140 and At5g15650 orthologues are found on LG B8. Ks values of these duplicates were between 0.01 and 0.15, indicating that the associated duplication events were recent and specific to the B genome.

### Plastid genome divergence

A phylogeny was reconstructed for the six *Brassica* species of U's triangle from plastid *maturase K* (*matK*) gene sequences available in the NCBI database (www.ncbi.nlm.nih.gov/nuccore). Sequence of *A. thaliana matK* was included as an outgroup. The aligned set of *matK* sequences of the six *Brassica* species was 1,572 bp in length, which was 6 bp shorter than the *matK* sequence of *A*. *thaliana*. After removing gaps, pairwise distances were calculated by the Kimura two-parameter method [34], and a neighbour-joining tree was constructed. The allotetraploid *B. carinata* and its cytoplasm donor, the diploid species *B. nigra*, formed one lineage; allotetraploids *B. juncea* and *B. napus*, which acquired cytoplasm from *B. rapa*, and diploid species *B. rapa* and *B. oleracea* clustered together in another lineage (Figure S2). These results are in congruence with earlier studies reporting the presence of the two lineages Rapa/Oleracea and Nigra in the genus *Brassica* (Table S1) [23]–[27].

Results obtained with the *matK* gene were confirmed by similar analysis using another plastid gene, *ndhF* (NADH dehydrogenase subunit F). Partial gene sequences of the three *Brassica* diploid species and *B. napus* were downloaded from the NCBI database (www.ncbi.nlm.nih.gov/nuccore) and aligned with the 2,241-bp complete gene sequence of *A. thaliana*. The reading frame of partial sequences was determined using *A. thaliana* as a reference. The phylogenetic tree constructed from the 732-bp common aligned sequence set revealed two lineages, with *B. rapa*, *B. oleracea* and *B. napus* clustering into one lineage, and *B. nigra* comprising the other.

To estimate the divergence time of chloroplast genomes of *B. rapa* and *B. nigra*, the two progenitors of *B. juncea*, Ks values were calculated for *matK* and *ndhF* genes. Divergence times were estimated assuming a mutation rate of 1.7×10^−9^ substitutions per synonymous site per year previously calculated for Brassicaceae chloroplast DNA [35]. Based on the Ks value of 0.044 calculated for *matK*, Rapa/Oleracea and Nigra plastid lines were estimated to have diverged from one another 12.0 mya; using the Ks value of 0.036 calculated for *ndhF*, a divergence time of 11.6 mya was inferred for these lineages. The calculated plastid genome divergence time for *B. rapa* and *B. nigra*, approximately 12 mya, coincides with the estimated nuclear divergence time of the three progenitors of present-day *Brassica* species A and B genomes.

## Discussion

The best evidence for independent origins of A and B genomes is derived from divergence times estimated from levels of synonymous nucleotide sequence divergence in plastid and nuclear genes. The evidence presented here, as well as extensive data available from earlier studies (Table S1), clearly reveals that A and B genomes represent two independent plastid lineages in tribe Brassiceae—the Rapa/Oleracea lineage and the Nigra lineage. Separation of these two lineages dates back to around 12 mya based on Ks values for plastid *matK* and *ndhF* gene sequences and the plastid DNA mutation rate estimate given by Koch et al. [35].

Previous research involving mapping and *in situ* hybridization have clearly established that *Brassica* A, B and C genomes are triplicated genomes [10]–[12], [14], [15]. If the three constituent genomes of *B. rapa* (AA) are represented as X, Y and Z and those of *B. nigra* (BB) as X′, Y′ and Z′, our synonymous nucleotide substitution data indicate that the X-X′, Y-Y′ and Z-Z′ (the A and B genome homoeologues in *B. juncea*) split occurred around 7 mya, and that the paralogues X-Y-Z and X′-Y′-Z′ separated about 12 mya. Ks values between the paralogues of the A genome, X-Y-Z, and the paralogues of the B genome, X′-Y′-Z′ are significantly different from levels of divergence between X-X′, Y-Y′ and Z-Z′ (Table 2).

**Figure 3:**
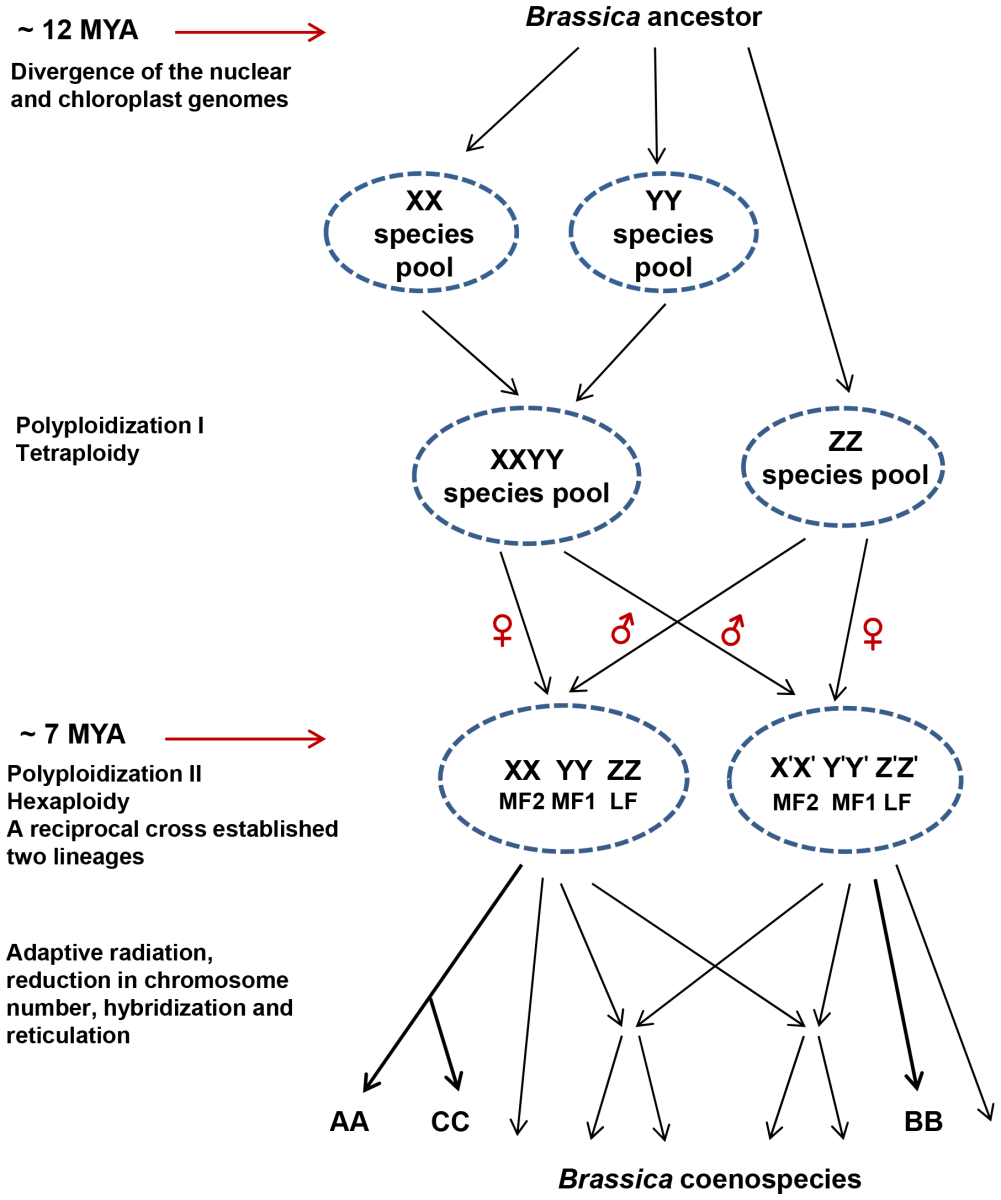
A model depicting the evolutionary events leading to divergence of the Rapa/Oleracea and Nigra lineages of Brassiceae. Length of the arrows does not represent evolutionary distance.

Based on the rate of synonymous substitutions among R-block nuclear genes and the overwhelming evidence supporting plastid Rapa/Oleracea and Nigra lineages, we propose the following model to explain the evolution of A and B genomes (Figure 3):

- The *Brassica* lineage split from the *Arabidopsis* lineage around 15 mya. Other studies have dated this separation to anywhere between 14.5 to 20 mya [36]–[38]. Nevertheless, all reported estimates show that the split between the *Brassica* and *Arabidopsis* lineages happened earlier than diversification of the *Brassica* lineage.
- The *Brassica* progenitor evolved into a number of species. In Figure 3, we show only three—X, Y and Z—to explain A and B genome evolution. It may be more appropriate, however, to describe X, Y and Z as X, Y and Z species pools.
- X, Y and Z have similar Ks values, and are therefore of similar antiquity. Based on gene fractionation patterns in *B. rapa*, it has been proposed recently that X and Y first came together to form an allotetraploid, followed by entry of the third genome, Z [16], [17]. Our model (Figure 3) agrees with this sequence of events.
- Recently generated *B. rapa* nuclear genome sequence data reveals that the Z genome is the least fractionated (LF), X is the most fractionated (MF2) and Y is medium fractionated (MF1) [16]. Our model agrees with this fractionation pattern.
- A minimum of two independent genome triploidization events, involving reciprocal crosses between an XY tetraploid and a Z diploid, and thereby establishing two plastid lineages, must have occurred about 7 mya. The ages of the plastid lineages date back to the independent formation of X, Y and Z species pools, approximately 12 mya.
- Stochastically, two reciprocal crosses would not survive in a panmictic population. If one cross had a selective advantage over the other, the latter would be rapidly eliminated. The two reciprocal crosses leading to the establishment of hexaploids (XXYYZZ) must have therefore occurred under reproductive isolation, and may have involved somewhat differentiated X, Y and Z species pools.
- Our model allows for gene fractionation [16], [17], chromosomal rearrangement [18], [39], hybridization and reticulation [3] during subsequent evolution of tribe Brassiceae (Figure 3).

A number of studies on family Brassicaceae have calculated Ks values and divergence times using different set of nuclear genes (Table S7). All the studies show that Arabidopsis-Brassica split has more antiquity than divergence within the tribe Brassiceae. The evolutionary divergence between the ancestral genomes—X, Y, Z—that took part in genome triplication has been reported to be more than divergence within each genome i.e between X-X′, Y-Y′, Z-Z′ (Table S7). Divergence time figures show the same trend in all the studies but have been interpreted to propose a single event of hexaploidy followed by divergence of the Nigra and Rapa/Oleracea lineages. This does not fit with the antiquity of plastid genome divergence of 12 mya calculated using the synonymous mutation rate estimates given by Koch et al. [35]. The model presented here reconciles both nuclear and plastid genome divergence times (Figure 3).

*Brassica* species have poor reproductive barriers, and hybrids could have readily formed at hexaploidy and subsequently between the descendants. Cytogenetic studies have clearly demonstrated the high intercrossability of *Brassica* species and significant pairing between homoeologous chromosomes [40]–[43]. As a consequence, a large number of species belonging to the tribe Brassiceae have been referred to as *Brassica* coenospecies.

A study of the Brassiceae ***U*** polyploidization event clearly demonstrates that reciprocal crosses can occur and survive. *Brassica napus* (AACC) and *B. juncea* (AABB) have A-genome (*B. rapa*) plastid genomes, whereas *B. carinata* (BBCC) has a B-genome (*B. nigra*) plastid genome [44]. The three polyploids originated in isolation. Further, sufficient data exists from plastid and nuclear genome studies of *B. napus* lines to suggest that this allopolyploid had two or more independent origins [9], [45]. Thus, the ***U*** polyploidization event involved both reciprocal and multiple crossing events. It is therefore entirely plausible that the ***b*** event involved multiple crosses that included reciprocal crossing.

The main difference between ***b*** and ***U*** events is the chromosomal number of the resulting allopolyploids. The ***U*** event gave rise to strict allotetraploids with chromosome numbers exactly equal to the sum of any two of the three U-triangle species—*B. rapa* (AA), *B. oleracea* (CC) and *B. nigra* (BB). In comparison, the ***b*** polyploidization event led to the evolution of mesoploids with chromosome numbers much reduced from the initial hexaploid chromosome number (2*n* = 6*x* = 42) [18].

Does the R-block gene fractionation pattern reported in this study support the hypothesis of independent origin of the two mesoploid genomes A and B? The six R blocks studied here (three from the A genome and three from the B genome) show very high gene collinearity and a conserved gene fractionation pattern with respect to the R block of *A. thaliana* and homoeologous LGs. The fractionation pattern is different between *B. rapa* and *B. nigra* in only seven of the 104 *Arabidopsis* genes (Figure 1). Gene redundancy exists in all these cases, as paralogues are available. Thus differential gene content could be explained either way—as evidence of independent origin or gradual gene loss over time.

Many gene duplications are specific to the B genome. Because these duplicated genes are of relatively recent origin, they do not provide any support for independent genome origins. The duplicated genes common to both A and B genomes may have either independent origins or shared lineages, and therefore are not rigorous evidence for the two-independent-lineage hypothesis. An interesting observation is the presence of many reading frames on LG B2 (MF2-R) and LG B8 (LF-R). These CDSs are unique to the B genome. Such unique CDSs were also observed on LG A2 (MF2-R) and LG A10 (LF-R) (Table 1). Some of these CDSs show identity with genes from distantly related plant families. It is possible that these genes were present in the putative Brassica ancestor but have been selectively lost in some of the descendants which constituted diverged species pools X, Y and Z. The differential loss of these CDS can be cited as an evidence for independent origin of A and B lineages.

A third piece of evidence can be gleaned from block arrangements on the A, B and C genomes. Based on the arrangement of 24 genomic blocks [13], some previous mapping studies using genic markers [12], [14] uncovered identical block arrangements between three LGs of A and C genomes (A1–C1, A2–C2 and A3–C3) and significant similarity between three LGs of the A and B genomes (A4–B4, A5–B5 and A6–B6). Our recent mapping work (unpublished) using transcriptome-based SNPs, however, has revealed much more extensive reshuffling on most LGs of the B genome. None of the LGs of the B genome seem to have the same block arrangement as those of the A genome. These differences are further suggestive of independent A and B genome origins.

Based on the divergence times estimated from synonymous base substitution rates in nuclear and plastid genes and their somewhat similar gene fractionation patterns, we conclude that reciprocal crossing between similar genomes at the time of the ***b*** event most plausibly explains the origin of *Brassica* A and B genomes. A recently published phylogenetic analysis of 89 Brassiceae species using sequences from four plastid intergenic regions—*rpl32-trnL, atpI-atpH, psbD-trnT* and *ycf6-psbM*—uncovered eight lineages: Rapa-Oleracea, Savignya, Nigra, Cakile, Crambe, Henophyton, Zilla and Vella [27]. Data was analysed by three different algorithms—maximum parsimony, maximum likelihood and Bayesian inference; all three methods yielded similar results confirming the eight independent lineages. Just like the Rapa/Oleracea and Nigra lineages, these additional lineages may also have resulted from independent triplication events involving species that were descendants of a putative ancestral species. Thus even though tribe Brassiceae is monophyletic – hexaploidization, the ***b*** event, was not a single event but a number of independent events involving hybridization between closely related or somewhat divergent species.

## Materials and Methods

### Plant materials and *B. juncea* BAC libraries

BAC library development was performed by Amplicon Express (Pullman, Washington, USA) using the *B. juncea* East European line Heera. Partial genomic digests of nuclear DNA were cloned into a pECBAC1 vector at a *Bam*HI site and into a pCC1BAC vector at a *Hind*III site for the construction of two independent BAC libraries. PCR screening of the BAC libraries was carried out using the Matrix Pool and Superpool strategy of Amplicon Express (http://ampliconexpress.com/products-services/screening-services/pools-and-superpools). Each *B. juncea* BAC library consisted of 55,296 clones with an average insert size of 120–130 kb distributed over 144 384-well plates, thus representing approximately five genomic equivalents of the *B. juncea* haploid genome estimated to be 1,068 Mb in size [46].

### Screening of BAC libraries

Dominant or co-dominant IP markers [14] specific to the target R-block region and the Heera line were used to screen the BAC libraries. Superpools were PCR-screened, with DNA from parents Varuna and Heera included as positive controls. A second PCR round for the specific superpool(s) identified in the first PCR round was then performed on the matrix pools. PCR conditions were the same as those previously used for genetic mapping of the IP markers onto a *B. juncea* linkage map [14]. The identified clones were streaked on LB agar plates, with five isolated colonies from each clone selected for plasmid isolation. Plasmid isolation was carried out using 3-ml LB cultures by the standard alkaline lysis method [47]. Clone confirmation of the isolated plasmid DNA was performed by PCR amplification of the IP marker region; the *Hind*III digestion pattern of the plasmid DNA was checked to detect any contamination.

### Construction of contigs across six R blocks in *B. juncea*

To construct a contig across the targeted region of a particular R block, anchor BACs were initially identified using IP markers mapped to that region. The gene span of the BAC clone was determined by amplification of the BAC DNA using primers designed from genes upstream and downstream of the IP marker used to select that clone. In addition, *B. juncea*, *B. rapa* and *B. nigra* DNAs were included as controls. To extend the contigs into regions where mapped markers were not available, suitable primers amplified by the last identified BAC clone in that region were used to screen more BAC clones. Because each LG's amplification pattern was unique in terms of fragment size, the correct assignment of new BACs to a particular region was confirmed by comparing their amplification patterns in the overlapping region with those of previous BACs.

Suitable mapped markers were not available in the target region of LG A3. Amplification with the IP primer for the target R-block region of *A. thaliana* gene At5g15320 generated four bands, three of which were confirmed to belong to LGs A10, B8 and B3. Based on gene fractionation patterns, which are usually identical for homoeologous LGs, the fourth band was assumed to belong to LG A3. This band was sequenced and BLASTN-searched against the A3 chromosome sequence of *B. rapa* [48]. Based on sequence similarities, the fourth band was confirmed to belong to chromosome A3 of *B. rapa*. This primer was then used for BAC clone screening to construct the *B. juncea* LG A3 contig.

### Sequencing of BAC clones and sequence analysis

Solexa-based next-generation sequencing of BAC clones was carried out by Amplicon Express. Annotation of the assembled scaffolds for the six R blocks was performed using the Brassica BAC annotation pipeline (http://brassica.nbi.ac.uk/annotate.html). Annotation details are available by scaffold-ID searching at http://brassica.nbi.ac.uk/cgi-bin/gbrowse/diy_brassica.

### Estimation of Ks and genome divergence times

The rate of synonymous substitutions (Ks value) was estimated by computing pairwise distances using the Nei-Gojobori [49] method as implemented in MEGA5. Differences among Ks values for the six orthologue comparisons (AT-BJ), six paralogue comparisons (three each in A and B genome) and three homoeologue comparisons (between A and B genomes) were statistically analysed using single-factor ANOVA as implemented in Microcal Origin version 6.0 (Microcal, Northampton, MA, USA). Significance of variation between mean Ks values for orthologue divergence (AT-BJ), paralogue divergence (within A and B genomes) and homoeologue divergence (between A and B genomes) was analysed statistically by unpaired Student's *t*-tests using Microcal Origin. For both statistical analyses, *p*<0.05 was considered to be statistically significant. Nuclear genome divergence times were calculated from Ks values using the equation T = Ks/(2×[1.5×10^−8^]), where 1.5×10^−8^ substitutions per site per year is the synonymous mutation rate [21].

Chloroplast divergence times were calculated by the same formula used for nuclear genome divergence times by adopting the synonymous mutation rate of 1.7×10^−9^ substitutions per site per year [35].

### Phylogenetic analyses

To reconstruct a nuclear genome phylogeny, alignments of 11 nuclear genes retained across all six R blocks were concatenated using the Phyutility software program [50]. A neighbour-joining tree was constructed using MEGA5 taking into consideration the synonymous base substitution rates and the distances were computed by Kimura two-parameter method [34]. The significance of nodes was tested by bootstrap analysis with 1,000 replicates.

Plastid genome phylogenies were reconstructed separately from full-length *matK* and partial *ndhF* sequences downloaded from the NCBI database (www.ncbi.nlm.nih.gov/nuccore) for the diploid and allotetraploid *Brassica* species and from the TAIR database (www.arabidopsis.org) for *A. thaliana*. Sampled taxa and their GenBank/TAIR accession numbers are as follows: for *matK* sequences, *B. rapa* (NC_015139), *B. nigra* (AB354272.1), *B. oleracea* (AB354271), *B. napus* (JF807904), *B. juncea* (AB354274.1), *B. carinata* (AB354275.1) and *A. thaliana* (ATCG00040), and for *ndhF* sequences, *B. rapa* (DQ200044), *B. nigra* (DQ200031), *B. oleracea* (AF064647), *B. napus* (DQ200041) and *A. thaliana* (ATCG01010). Neighbour-joining trees were constructed in MEGA5 based on nucleotide substitutions, with distances estimated by the Kimura two-parameter method [34].

## Supporting Information

Figure S1 **Contigs assembled using overlapping BAC clones in the target R block region in the six linkage groups of** ***B. juncea*** **.** Mapping information is from Panjabi et al. [14]. BACs with H prefix are from *Hind*III library and those with B prefix are from *Bam*HI library. Colour code in the contigs constructed from overlapping BAC clones indicates the *A. thaliana* genes used to design primers to pick up the corresponding BAC clone.
(PPTX)

Figure S2 **Neighbour joining tree of six** ***Brassica*** **crop species constructed based on the chloroplast gene** ***matK*** **sequence.** The numbers on the nodes represent percentage from bootstrap analysis of 1000 replicates.
(PPTX)

Table S1 **Assignment of different species of tribe Brassiceae to the Rapa/Oleracea or the Nigra lineages based on chloroplast genome analysis.** Haploid chromosome number of the species as well as the species in which genome triplication has been shown by *in situ* hybridization are also recorded [51], [52].
(XLSX)

Table S2 **List of BAC clones identified for the target region in six R blocks of** ***B. juncea*** **.** BACs with H prefix are from *Hind*III library and those with B prefix are from *Bam*HI library.
(DOCX)

Table S3 **List of the BAC clones sequenced for the targeted regions of the six R blocks of** ***B. juncea*** **.**
(DOCX)

Table S4 **Comparison of gene organisation in the targeted R block regions on six linkage groups of** ***B. juncea*** **with syntenous regions of** ***B. rapa*** **and** ***A. thaliana*** **.**
(XLSX)

Table S5 **BLASTX analysis of the coding sequences (CDS) in the** ***B. juncea*** **R blocks from noncollinear regions.**
(DOCX)

Table S6 **List of 11 genes considered for the nuclear divergence analysis with their assigned functions in** ***A. thaliana*** **.**
(XLSX)

Table S7 **Nuclear genome divergence time estimates in** ***Arabidopsis*** **/** ***Brassica*** **evolution from different studies** [53]–[56] **.**
(XLSX)
