# Supplementary material for: Two Plastid DNA Lineages—Rapa/Oleracea and Nigra—within the Tribe Brassiceae Can Be Best Explained by Reciprocal Crosses at Hexaploidy: Evidence from Divergence Times of the Plastid Genomes and R-Block Genes of the A and B Genomes of Brassica juncea
Source: PLoS One. 2014 Apr 1;9(4):e93260. doi: 10.1371/journal.pone.0093260 (PMC3972200; doi:10.1371/journal.pone.0093260)
Supplement: Table S2 — List of BAC clones identified for the target region in six R blocks of B. juncea . BACs with H prefix are from HindIII library and those with B prefix are from BamHI library. (DOCX) [file pone.0093260.s004.docx]

**Table S2 List of BAC clones identified for the target region in six R blocks of *B. juncea*.**

BACs with H prefix are from *Hind*III library and those with B prefix are from *Bam*HI library.

| ***A. thaliana* genes used for BAC clone identification** | **BAC Clones** | **Gene Span** |
| --- | --- | --- |
| **A2-R Block** | | |
| At5g14720, At5g14790 | H-25-I24 | At5g14720 to At5g15050 |
|  | H-36-N17 | At5g14720 to At5g15230 |
|  | H-102-K10 | At5g14720 to At5g14950 |
|  | H-132-I12 | At5g14440 to At5g14890 |
|  | H-138-M11 | At5g14660 to At5g14720 |
| A5g15200 | B-112-K11 | At5g14940 to At5g15200 |
|  | B-72-K08 | At5g15150 to At5g15580 |
|  | B-87-F14 | At5g15150 to At5g15550 |
| A5g15400 | B-79-M11 | At5g15390 to At5g15750 |
|  | B-80-J17 | At5g15390 to At5g15650 |
| At5g15680, At5g15930 | H-35-N01 | At5g15400 to At5g15930 |
|  | H-59-E09 | At5g15540 to At5g15930 |
|  | H-30-O19 | At5g15550 to At5g15930 |
|  | B-90-B22 | At5g15400 to At5g15750 |
|  | B-132-E17 | At5g15470 to At5g15930 |
| **B2-R Block** | | |
| At5g14720 | B-112-P06 | At5g14540 to At5g15050 |
|  | B-25-P09 | At5g14540 to At5g14740 |
| At5g15200 | B-108-N05 | At5g15150 to At5g15470 |
| At5g15230 | H-36-L05 | At5g15050 to At5g15400 |
|  | H-142-A12 | At5g15200 to At5g15390 |
| At5g15580 | B-58-K22 | At5g15200 to At5g15700 |
|  | B-137-I24 | At5g15230 to At5g15700 |
| At5g15860 | B-96-J12 | At5g15390 to At5g15860 |
|  | B-100-M24 | At5g15540 to At5g15860 |
|  | B-84-A17 | At5g15700 to At5g15860 |
| **A3-R block** | | |
| At5g14920 | B-92-B23 | At5g14320 to At5g15080 |
|  | B-129-C12 | At5g14520 to At5g15120 |
| At5g15320 | H-5-P05 | At5g15200 to At5g15880 |
|  | H-8-I23 | At5g15120 to At5g15750 |
|  | H-62-L09 | At5g15150 to At5g15650 |
|  | H-71-E05 | At5g15200 to At5g15550 |
|  | H-83-H13 | At5g14910 to At5g15740 |
|  | B-108-P14 | At5g15020 to At5g15470 |
| **B3-R block** | | |
| At5g14640 | B-53-J21 | At5g14640 to At5g15120 |
|  | B-61-K03 | At5g14640 to At5g15120 |
|  | B-114-A08 | At5g14640 to At5g15120 |
|  | B-45-P11 | At5g14640 to At5g15020 |
|  | B-72-M09 | At5g14640 to At5g14930 |
|  | B-24-E05 | At5g14640 to At5g14920 |
| At5g15230 | B-23-P05 | At5g14910 to At5g15350 |
| At5g15680 | B-43-I8 | At5g15350 to At5g15880 |
|  | B-76-H14 | At5g15350 to At5g15880 |
|  | B-131-O21 | At5g15350 to At5g15880 |
|  | H-17-M06 | At5g15320 to At5g15740 |
|  | B-89-F17 | At5g15310 to At5g15880 |
|  | B-116-O23 | At5g15310 to At5g15880 |

| ***A. thaliana* genes used for BAC clone identification** | **BAC Clones** | **Gene Span** |
| --- | --- | --- |
| A10-R Block | | |
| At5g14660 | H-133-F11 | At5g14460 to At5g14780 |
| At5g15150 | H-47-I13 | At5g15150 to At5g15300 |
|  | H-91-M20 | At5g14930 to At5g15400 |
|  | H-96-A24 | At5g15150 to At5g15400 |
|  | H-119-N06 | At5g14720 to At5g15320 |
|  | H-7-N09 | At5g14650 to At5g15150 |
| At5g15320 | H-74-B02 | At5g15290 to At5g15550 |
|  | H-52-P23 | At5g15120 to At5g15410 |
|  | H-107-E24 | At5g14720 to At5g15470 |
|  | H-122-C17 | At5g14940 to At5g15410 |
|  | H-126-N08 | At5g15120 to At5g15330 |
| At5g15840 | B-125-C18 | At5g16070 to At5g15800 |
|  | B-54-H11 | At5g15880 to At5g15650 |
|  | B-42-J04 | At5g15840 to At5g15650 |
|  | B-92-C07 | At5g15840 to At5g15540 |
|  | B-70-I23 | At5g15840 to At5g15530 |
| B8-R Block | | |
| At5g14660 | B-95-E14 | At5g14180 to At5g14720 |
|  | B-115-A16 | At5g14370 to At5g14780 |
| At5g14870 | H-64-P17 | At5g14660 to At5g14890 |
|  | H-125-M11 | At5g14700 to At5g14940 |
| At5g14940 | H-52-O16 | At5g14930 to At5g14940 |
|  | B-128-K11 | At5g14890 to At5g15150 |
| At5g15320, At5g15330 | B-10-C01 | At5g15370 to At5g15580 |
|  | B-30-M09 | At5g15330 to At5g15580 |
|  | B-67-H22 | At5g15290 to At5g15550 |
|  | B-116-K20 | At5g15200 to At5g15390 |
|  | H-2-I21 | At5g15200 to At5g15530 |
|  | H-11-F03 | At5g15200 to At5g15550 |
|  | H-14-E10 | At5g15200 to At5g15320 |
|  | H-52-F07 | At5g15200 to At5g15320 |
|  | H-71-M24 | At5g15290 to At5g15650 |
|  | H-80-I08 | At5g15310 to At5g15550 |
|  | H-121-F22 | At5g14920 to At5g15530 |
| At5g15400 | B-97-O02 | At5g15200 to At5g15540 |
|  | B-144-P02 | At5g15320 to At5g15540 |
| At5g15650 | B-5-E04 | At5g15650 to At5g15800 |
|  | B-54-H11 | At5g15650 to At5g15840 |
|  | B-83-P09 | At5g15650 to At5g15800 |
|  | B-96-A01 | At5g15540 to At5g15740 |
