# Supplementary material for: Two Plastid DNA Lineages—Rapa/Oleracea and Nigra—within the Tribe Brassiceae Can Be Best Explained by Reciprocal Crosses at Hexaploidy: Evidence from Divergence Times of the Plastid Genomes and R-Block Genes of the A and B Genomes of Brassica juncea
Source: PLoS One. 2014 Apr 1;9(4):e93260. doi: 10.1371/journal.pone.0093260 (PMC3972200; doi:10.1371/journal.pone.0093260)
Supplement: Table S3 — List of the BAC clones sequenced for the targeted regions of the six R blocks of B. juncea . (DOCX) [file pone.0093260.s005.docx]

**Table S3 List of the BAC clones sequenced for the targeted regions of the six R blocks**

**of *B. juncea.***

| **BAC clones** | **Scaffolds** | **Size (Kb)** |
| --- | --- | --- |
| Contig Assembly for A2 R block | | |
| H-132-I12 | A2-scaffold_0 | 367 |
| H-36-N17 |  |  |
| B-87-F14 |  |  |
| H-35-N01 |  |  |
| Contig Assembly for B2 R block | | |
| B-112-P06 | B2,scaffold_0 | 432 |
| H-36-L05  B-58-K22  B-96-J12 | B2-scaffold-0-1*  B2-scaffold-0-2*  B2-scaffold-0-3* |  |
|  |  |  |
|  |  |  |
| Contig Assembly for A3 R block | | |
| H-5-P05 | A3-scaffold_9 | 176 |
| H-83-H13 | A3-scaffold_28 | 93 |
| B-129-C12 |  |  |
| Contig Assembly for B3 R block | | |
| B-53-J21 | B3-scaffold_35 | 53 |
| B-23-P05 | B3-scaffold_71 | 10 |
| B-116-O23 | B3-scaffold_64 | 12 |
|  | B3-scaffold_48 | 23 |
|  | B3-scaffold_47 | 28 |
|  | B3-scaffold_18 | 113 |
| Contig Assembly for A10 R block | | |
| H-133-F11 | A10-scaffold_7 | 177 |
| H-107-E24 | A10-scaffold_17 | 116 |
| H-74-B02 | A10-scaffold_5 | 208 |
| B-70-I23 |  |  |
| B-125-C18 |  |  |
| Contig Assembly for B8 R block | | |
| B-115-A16 | B8-scaffold_6 | 217 |
| H-125-M11 | B8-scaffold_0 | 445 |
| B-128-K11 |  |  |
| H-121-F22 |  |  |
| H-71-M24 |  |  |
| B-96-A01 |  |  |
| B-54-H11 |  |  |

* - The three scaffolds represent overlapping regions of a single scaffold - B2, scaffold_0.
