# Supplementary material for: Two Plastid DNA Lineages—Rapa/Oleracea and Nigra—within the Tribe Brassiceae Can Be Best Explained by Reciprocal Crosses at Hexaploidy: Evidence from Divergence Times of the Plastid Genomes and R-Block Genes of the A and B Genomes of Brassica juncea
Source: PLoS One. 2014 Apr 1;9(4):e93260. doi: 10.1371/journal.pone.0093260 (PMC3972200; doi:10.1371/journal.pone.0093260)
Supplement: Table S5 — BLASTX analysis of the coding sequences (CDS) in the B. juncea R blocks from noncollinear regions. (DOCX) [file pone.0093260.s007.docx]

**Table S5 BLASTX analysis of the coding sequences (CDS) in the *B. juncea* R blocks from noncollinear regions.**

The three color codes indicate the similarity of the CDS with different types of proteins.

Comparison of the CDS with transcriptome sequence assembly of *B. rapa*, *B. nigra* and *B. juncea* was done using BLASTN.

* - CDS detected in the transcriptome of *B. rapa* and *B. juncea* A genome

** - CDS detected in the transcriptome of *B. nigra*  B genome

***- CDS detected in the transcriptome of both A (*B. rapa* and *B. juncea*) and B (*B. nigra*) genomes

| **Sr. no.** | **Predicted gene ID** | **% Similarity** | **% Coverage** | **Similar to proteins in plant species** | **Similar to proteins** |
| --- | --- | --- | --- | --- | --- |
| **CDS showing similarity with proteins coded by *B. rapa* genes from other blocks** | | | | | |
| 1 | A2-sc-0.40^***^ | 96.09 | 100.00 | *B. rapa* | flavodoxin-like quinone reductase 1 |
| 2 | A2-sc-0.8^*^ | 100 | 35.14 | *B. rapa* | Esterase/lipase/thioesterase family protein |
| 3 | A3-sc-9.1^***^ | 81.48 | 10.80 | *B. rapa* | F-box family protein |
| 4 | A3-sc-9.48^*^ | 42.7 | 34.7 | *B. rapa* | Expressed protein AT3G06435 |
| 5 | A3-sc-9.6+sc-9.7^***^ | 76.27 | 34.50 | *B. rapa* | RabGAP/TBC domain-containing protein |
| 6 | A10-sc-5.28^*^ | 48.39 | 30.4 | *B. rapa* | Transcription factor |
| 7 | A10-sc-5.41 | 50.5 | 99.8 | *B. rapa* | Putative uncharacterized protein F2J7.11 |
| 8 | A10-sc-7.24^***^ | 99.33 | 100.00 | *B. rapa* | U-box domain-containing protein |
| 9 | A10-sc-7.33^***^ | 94.12 | 40.24 | *B. rapa* | ATP binding / kinase/ protein serine/threonine kinase |
| 10 | A10-sc-7.34^***^ | 98.25 | 5.65 | *B. rapa* | Heat shock protein-related |
| 11 | A10-sc-7.37^*^ | 57.14 | 42 | *B. rapa* | VOZ1, ATVOZ1; VOZ1 (vascular plant one zinc finger protein); transcription activator |
| 12 | A10-sc-17.16^***^ | 62.53 | 99.6 | *B. rapa* | Protein kinase family protein |
| 13 | A10-sc-5.40 | 47.69 | 100.00 | *B. rapa* | Zinc knuckle (CCHC-type) family protein |
| 14 | A10-sc-5.47^*^ | 42.19 | 35.75 | *B. rapa* | DNA N-glycosylase/ DNA-(apurinic or apyrimidinic site) lyase/ protein binding |
| 15 | B2-sc-0-1.17^**^ | 25 | 30.4 | *B. rapa* | Phototropic-responsive NPH3 protein-related |
| 16 | B2-sc-0-2.56^**^ | 69.44 | 72.97 | *B. rapa* | peptidyl-prolyl cis-trans isomerase |
| 17 | B2-sc-0-2.51^***^ | 67.86 | 26.16 | *B. rapa* | Unknown protein |
| 18 | B2-sc-0-2.5 | 39.8 | 31.1 | *B. rapa* | Nucleic acid binding / ribonuclease H |
| 19 | B2-sc-0-2.42 | 42.86 | 30.4 | *B. rapa* | protein binding / zinc ion binding |
| 20 | B2-sc-0-2.28^**^ | 56.25 | 91.43 | *B. rapa* | Ubiquitin-protein ligase |
| 21 | B2-sc-0-2.14 | 32.96 | 62.6 | *B. rapa* | Unknown protein |
| 22 | B2-sc-0-2.13^**^ | 47.3 | 13.05 | *B. rapa* | stress-inducible protein, putative |
| 23 | B2-sc-0-1.38^**^ | 53.24 | 20.87 | *B. rapa* | zinc knuckle (CCHC-type) family protein |
| 24 | B2-sc-0-1.37^***^ | 50.57 | 82.86 | *B. rapa* | P-P-bond-hydrolysis-driven protein transmembrane transporter |
| 25 | B2-sc-0-1.36^**^ | 49.12 | 43.1 | *B. rapa* | CTP synthase, putative / UTP--ammonia ligase, putative |
| 26 | B2-sc-0-1.35^***^ | 87.01 | 16.67 | *B. rapa* | Mid chain alkane hydroxylase/ oxygen bindingm |
| 27 | B2-sc-0-1.33^**^ | 82.33 | 100.00 | *B. rapa* | F-box family protein |
| 28 | B3-sc-35.3^*^ | 56.08 | 100.00 | *B. rapa* | Disease resistance protein (TIR-NBS-LRR class), putative |
| 29 | B3-c-512.2^**^ | 76.32 | 10.86 | *B. rapa* | Invertase/pectin methylesterase inhibitor family protein |
| 30 | B8-sc-0.114 | 44.44 | 58.38 | *B. rapa* | RNA recognition motif (RRM)-containing protein |
| 31 | B8-sc-0.112 | 30.21 | 49.3 | *B. rapa* | F-box family protein |
| 32 | B8-sc-0.109^**^ | 62.69 | 16.90 | *B. rapa* | Agent domain-containing protein |
| 33 | B8-sc-0.99^***^ | 40.58 | 22.6 | *B. rapa* | GTP-binding protein |
| 34 | B8-sc-0.87 | 50.6 | 19.8 | *B. rapa* | cysteine-type peptidase |
| 35 | B8-sc-0.82^**^ | 70 | 40.00 | *B. rapa* | Unknown protein |
| 36 | B8-sc-0.71^**^ | 68.09 | 38.84 | *B. rapa* | Encodes a Plant thionin family protein |
| 37 | B8-sc-0.49 | 50 | 11.2 | *B. rapa* | Cysteine type peptidase |
| 38 | B8-sc-0.48^**^ | 36.43 | 13.8 | *B. rapa* | Cysteine type peptidase |
| 39 | B8-sc-0.32 | 40 | 24 | *B. rapa* | DNA binding protein |
| 40 | B8-sc-0.31^**^ | 62.1 | 34.8 | *B. rapa* | DNA binding |
| 41 | B8-sc-0.18^**^ | 73.33 | 60.61 | *B. rapa* | Unknown protein |
| 42 | B8-sc-0.17^**^ | 50.6 | 58.0 | *B. rapa* | Cysteine protease inhibitor family protein |
| 43 | B8-sc-0.16^**^ | 69.81 | 56.1 | *B. rapa* | Protein kinase family protein |
| **CDS showing similarity with proteins from genera other than *B. rapa*** | | | | | |
| 1 | A2-sc-0.28 | 48.4 | 14.5 | *A. thaliana* | F28K20.4 protein |
| 2 | A2-sc-0.30 | 34.57 | 39.3 | *Selaginella moellendorffii* | Putative uncharacterized protein |
| 3 | A2-sc-0.51 | 49.0 | 83.7 | *A. lyrata* | Putative uncharacterized protein |
| 4 | A3-sc-9.11^*^ | 27.66 | 30 | *Sorghum vulgare* | Putative uncharacterized protein |
| 5 | B2-sc-0-1.25^**^ | 43.1 | 13.8 | *B. oleracea* | Glutathione S-transferase |
| 6 | B2-sc-0-2.20^***^ | 50.36 | 85.31 | *Oryza sativa* | Putative helicase |
| 7 | B2-sc-0-2.22 | 42.55 | 37.7 | *Chlorella variabilis* | Putative uncharacterized protein |
| 8 | B2-sc-0-2.23 | 57.0 | 100.0 | *B. oleracea* | Putative uncharacterized protein |
| 9 | B2-sc-0-2.29 | 39.02 | 30 | *Jatropha curcas* | JHL17M24.3 protein |
| 10 | B2-sc-0-2.30 | 29.73 | 30.8 | *A. lyrata* | Malate dehydrogenase |
| 11 | B2-sc-0-2.31 | 27.5 | 34.6 | *A. thaliana* | AAF18631.1 |
| 12 | B2-sc-0-2.33 | 36.71 | 43.5 | *Volvox carteri* | Putative uncharacterized protein |
| 13 | B8-sc-0.59 | 35 | 38.5 | *Sorghum vulgare* | Putative uncharacterized protein |
| 14 | B8-sc-0.72 | 30.51 | 31.6 | *Oryza sativa subsp. japonica* | Putative uncharacterized protein |
| 15 | B8-sc-0.83 | 40.48 | 36.2 | *Oryza sativa subsp. japonica* | OSJNBa0065J03.16 protein |
| 16 | B8-sc-6.26^**^ | 35.14 | 30.8 | *Volvox carteri* | Putative uncharacterized protein |
| **CDS showing similarity with the transposon related proteins** | | | | | |
| 1 | A3-sc-9.26 | 43.3 | 10.4 | *A. thaliana* | LTR retroelement reverse transcriptase-like protein |
| 2 | A10-sc-17.11^*^ | 89.7 | 97.6 | *B. oleracea* | En/Spm-related transposon protein |
| 3 | B2-sc-0-1.34^**^ | 46.7 | 49.1 | *B. rapa* | Reverse transcriptase, putative / RNA-dependent DNA polymerase, putative |
| 4 | B2-sc-0-1.43^**^ | 65.1 | 16.2 | *B. rapa* | NRPD1B, DRD3, ATNRPD1B, DMS5, NRPE1; NRPD1B; DNA binding / DNA-directed RNA polymerase |
| 5 | B2-sc-0-2.10 | 44.9 | 31.2 | *B. rapa* | Reverse transcriptase, putative / RNA-dependent DNA polymerase, putative |
| 6 | B2-sc-0-2.15 | 54.1 | 100.0 | *A. thaliana* | RNA-directed DNA polymerase-like protein |
| 7 | B2-sc-0-2.3 | 59.8 | 16.0 | *A. thaliana* | RNA-directed DNA polymerase-like protein |
| 8 | B2-sc-0-2.32^**^ | 39.5 | 69.0 | *A. thaliana* | Gag/pol polyprotein |
| 9 | B2-sc-0-2.4 | 66.4 | 24.5 | *B. rapa* | Reverse transcriptase, putative / RNA-dependent DNA polymerase, putative |
| 10 | B2-sc-0-2.48^**^ | 66.18 | 10.18 | *B. rapa* | Reverse transcriptase, putative / RNA-dependent DNA polymerase, putative" |
| 11 | B2-sc-0-2.8 | 46.1 | 58.8 | *A. thaliana* | Non-LTR retroelement reverse transcriptase |
| 12 | B2-sc-0-2.9 | 55.9 | 64.6 | *A. thaliana* | RNA-directed DNA polymerase-like protein |
| 13 | B8-sc-0.33 | 59.2 | 44.4 | *A. thaliana* | HAT-element transposase |
| 14 | B8-sc-0.34 | 72.6 | 49.7 | *A. thaliana* | Putative TNP2-like transposon protein |
| 15 | B8-sc-0.35 | 45.65 | 32.3 | *A. thaliana* | HAT-element transposase |
| 16 | B8-sc-0.46^**^ | 35.6 | 20.0 | *A. thaliana* | Mutator-like transposase |
| 17 | B8-sc-0.47 | 50.4 | 39.9 | *A. thaliana* | Mutator-like transposase |
| 18 | B8-sc-0.58^**^ | 66.1 | 37.9 | *A. thaliana* | HAT-element transposase |
| 19 | B8-sc-0.66^**^ | 58.7 | 47.9 | *A. thaliana* | HAT-element transposase |
| 20 | B8-sc-0.75 | 40.0 | 12.8 | *A. thaliana* | non-LTR retroelement reverse transcriptase |
| 21 | B8-sc-0.84 | 66.0 | 45.7 | *A. thaliana* | Copia polyprotein |
| 22 | B8-sc-0.93 | 46.0 | 10.1 | *A. thaliana* | Retrotransposon reverse transcriptase |
| 23 | B8-sc-0.113^**^ | 38.16 | 40.34 | *A.thaliana* | Retroelement pol polyprotein-like |
